# Hierarchical Core/Shell NiCo_2_O_4_@NiCo_2_O_4_ Nanocactus Arrays with Dual-functionalities for High Performance Supercapacitors and Li-ion Batteries

**DOI:** 10.1038/srep12099

**Published:** 2015-07-01

**Authors:** Jinbing Cheng, Yang Lu, Kangwen Qiu, Hailong Yan, Jinyou Xu, Lei Han, Xianming Liu, Jingshan Luo, Jang-Kyo Kim, Yongsong Luo

**Affiliations:** 1 Key Laboratory of Advanced Micro/Nano Functional Materials, School of Physics and Electronic Engineering, Xinyang Normal University, Xinyang, P. R. China; 2 School of Material Science and Engineering, Hebei University of Technology, Tianjin, P. R. China; 3 College of Chemistry and Chemical Engineering, Luoyang Normal University, Luoyang, P. R. China; 4 Division of Physics and Applied Physics, School of Physical and Mathematical Sciences, Nanyang Technological University, Singapore; 5 Department of Mechanical and Aerospace Engineering, The Hong Kong University of Science and Technology, Clear Water Bay, Kowloon, Hong Kong, P. R. China

## Abstract

We report the synthesis of three dimensional (3D) NiCo_2_O_4_@NiCo_2_O_4_ nanocactus arrays grown directly on a Ni current collector using a facile solution method followed by electrodeposition. They possess a unique 3D hierarchical core-shell structure with large surface area and dual-functionalities that can serve as electrodes for both supercapacitors (SCs) and lithium-ion batteries (LIBs). As the SC electrode, they deliver a remarkable specific capacitance of 1264 F g^−1^ at a current density of 2 A g^−1^ and ~93.4% of capacitance retention after 5000 cycles at 2 A g^−1^. When used as the anode for LIBs, a high reversible capacity of 925 mA h g^−1^ is achieved at a rate of 120 mA g^−1^ with excellent cyclic stability and rate capability. The ameliorating features of the NiCo_2_O_4_ core/shell structure grown directly on highly conductive Ni foam, such as hierarchical mesopores, numerous hairy needles and a large surface area, are responsible for the fast electron/ion transfer and large active sites which commonly contribute to the excellent electrochemical performance of both the SC and LIB electrodes.

Developing efficient energy storage systems is an urgent requirement to meet the needs of modern society and ecological concerns^1^,^2^,^3^. Lithium-ion batteries (LIBs) and supercapacitors (SCs), as two major devices for electrochemical energy storage, have been receiving global attention because of their vital roles in our daily life^4^,^5^,^6^. Currently, tremendous efforts have been devoted to rational synthesis of advanced core/shell heterostructures with fascinating synergetic properties and multi-functionalities offered by various composite nanostructures. For example, Zhou *et al.* reported a three-dimensional (3D) CoO@PPy hybrid nanowire on Ni foam with outstanding pseudocapacitive performance that exhibited a remarkable areal specific capacitance (ASC) of 4.4 F cm^−2^ at 1 mA cm^−2^, nearly 4 times higher than 1.23 F cm^−2^ of the pristine CoO nanowire electrode^7^. Ternary NiCo_2_O_4_ composites have also attracted much attention because they are of low cost, environmentally benign, and naturally abundant as well as possess high theoretical capacitance. For instance, Lou *et al.*^8^ reported a solution synthesis of NiCo_2_O_4_ nanorods and nanosheets on carbon nanofibers, presenting excellent specific capacitances of 905 and 888.7 F g^−1^, respectively, at 2 A g^−1^. However, transition metal oxides including NiCo_2_O_4_ usually showed limited kinetics during the redox reaction as a result of their low electrical conductivities and relatively small surface areas^9^,^10^. Large volume changes and stresses commonly occur during the lithium insertion-exaction processes, resulting in pulverization of the electrodes and aggregation of electrode materials. This causes a large increase in contact resistance and significant capacity fade during cycling, thus limiting the commercial applications of these anode materials^11^,^12^. Tremendous efforts have been devoted to solve the problem of poor cycling of anode materials. One effective strategy is to find a suitable matrix to accommodate their volume change^13^,^14^. Various core/shell nanostructures such as semiconductor/semiconductor^15^, semiconductor/metal^16^, metal/metal^17^, metal oxide/metal oxide^18^, and metal oxide/conductive polymer^19^ have been explored, and exhibiting much better electrochemical performance in comparison with their bare counterparts.

Therefore, to satisfy the requirements of high specific capacitance, high specific capacity and durable structural stability, and to promote full utilization of the active materials for both SCs and LIBs, promising strategies include rational design of nano-architectured electrodes and hybridization of bespoke pseudocapacitive oxides. Among various forms of NiCo_2_O_4_ structures, hierarchical core/shell nanostructures make an excellent candidate for the construction of electrodes of high-performance SCs and LIBs. In this design, it is aimed that both the “core” and “shell” materials are to be effectively utilized to contribute to enhanced capacitance and capacity of the electrodes. We report a cost-effective and simple strategy to fabricate novel, hierarchical NiCo_2_O_4_@NiCo_2_O_4_ core/shell nanocactus arrays (NCAs) directly grown on Ni foam as a binder-free electrode for high-performance electrochemical energy storage devices. The smart electrode design offers several unique structural and materials features as follows. The large surface area arising from the hierarchical mesoporosity of the shell offers full exposure of the active material to the electrolyte^20^. Because of the porous shell structure, the electrolyte can easily penetrate into the inner region of the electrode, promoting the utilization of the active materials, both shell and core materials contributing to the electrochemical charge storage. Freestanding electrodes are prepared without adding conductive additives or polymer binders due to the presence of the highly conductive rigid Ni foam substrate, substantially reducing the “dead volume” in electrode materials^21^,^22^.

## Results and discussion

### Synthesis and characterization

**Figure 1:**
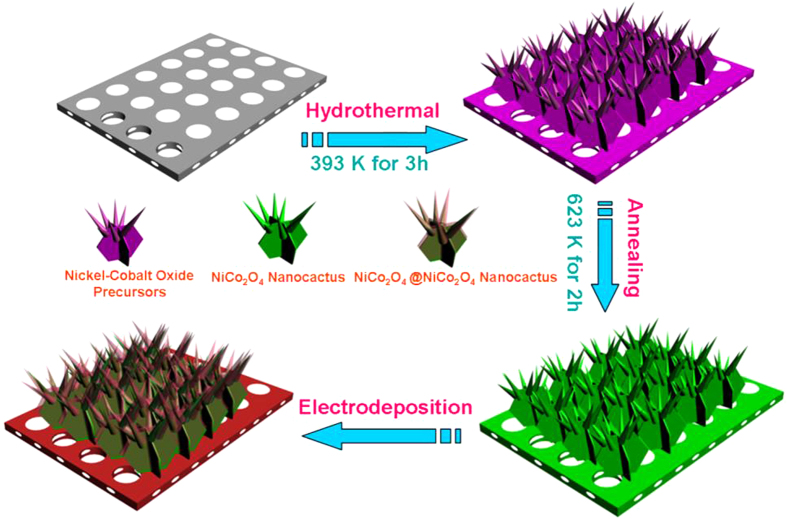
Schematic illustrating the fabrication processes of the NiCo_2_O_4_@NiCo_2_O_4_ core/shell nanocactus arrays.

Figure 1 schematically illustrates the fabrication procedure of the three-dimensional NiCo_2_O_4_@NiCo_2_O_4_ core/shell nanocactus arrays. First, NiCo_2_O_4_ nanocactus arrays were directly grown on a nickel foam substrate via a facile, hydrothermal process. Then, the as-prepared NiCo_2_O_4_ NCAs were subsequently annealed in air while maintaining the cactus shaped acicular structure. Finally, NiCo_2_O_4_@NiCo_2_O_4_ NCA core/shell structures were obtained through the growth of NiCo_2_O_4_ shell via an electrochemical deposition process.

**Figure 2:**
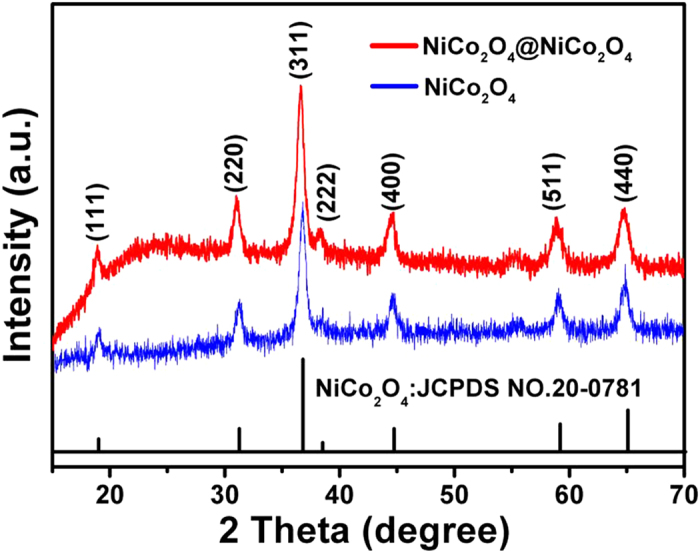
XRD patterns of NiCo_2_O_4_@NiCo_2_O_4_ and NiCo_2_O_4_ NCAs.

The crystallographic phase of the NiCo_2_O_4_@NiCo_2_O_4_ NCAs was identified by XRD, and the typical wide-angle diffraction patterns were shown in Fig. 2. They consisted of seven well-defined diffraction peaks that can be indexed into a cubic spinel NiCo_2_O_4_ crystalline structure (JCPDF card No. 20-0781). In general, the coexistence of Co and Ni in the oxide favors the formation of NiCo_2_O_4_^23^. The composition and structure of the NiCo_2_O_4_@NiCo_2_O_4_ NCAs were further confirmed by Raman analysis, as shown in Figure S1. Four prominent peaks were observed at 187, 477.8, 523.5 and 671.2 cm^−1^, assigning to the F_2g_, E_g_, F_2g_ and A_1g_ models of NiCo_2_O_4_, respectively. Only the Co-O and Ni-O vibrations were detected, indicating that the precursor cobalt/nickel carbonate hydroxide salts were completely transformed into oxides after calcinating at 350 ^o^C. These results are consistent with those reported previously^24^,^25^.

**Figure 3:**
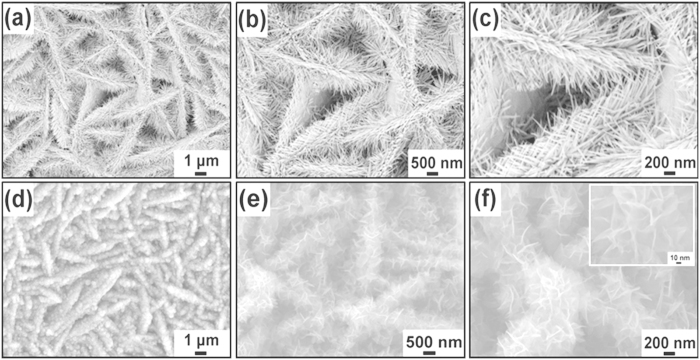
SEM images of(**a-c**) NiCo_2_O_4_ NCAs taken at low- and high-magnifications; (**d-f**) NiCo_2_O_4_@NiCo_2_O_4_ NCAs taken at different magnifications.

Figure 3a shows the SEM image of the as-synthesized well-ordered NiCo_2_O_4_ NCAs. It can be seen that large-scale, dense and aligned NiCo_2_O_4_ nanocactuses grow uniformly on the skeletons of Ni foam. The NCA structure was hierarchical, consisting of primary intermingled stems and secondary acicular needles emanated from the main stems, as shown by the high-magnification SEM images (Fig. 3b,c). The acicular needles were several tens of nanometer thick and more than 1 μm long. After the electrochemical deposition and annealing, a thin layer of NiCo_2_O_4_ flakes was covered on the surface of each NiCo_2_O_4_ nanocactus, forming a core/shell hierarchical structure (Fig. 3d). It can be found that the NiCo_2_O_4_ nanoflakes were porous and interconnected with each other (Fig. 3e,f), which was further confirmed by higher magnification SEM image (inset of Fig. 3f). The pores or voids inside the structure are beneficial to the electrolyte infiltration, and the interconnected nature enables fast ion and electron transportation.

**Figure 4:**
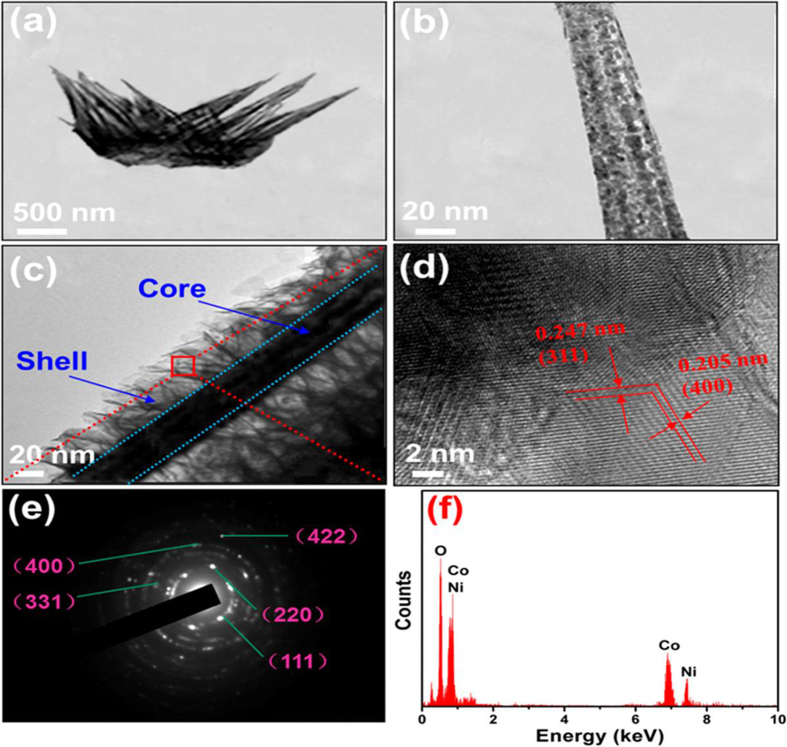
TEM images of(**a, b**) NiCo_2_O_4_ NCAs taken at low- and high-magnifications; (**c**) NiCo_2_O_4_@NiCo_2_O_4_ core/shell nanocactus; (**d, e**) HRTEM image and SAED pattern of the shell in (**c**); (**f**) EDS of the shell in (**c**).

More detailed information about the morphological and structural features of the as-synthesized NiCo_2_O_4_ and NiCo_2_O_4_@NiCo_2_O_4_ NCAs was obtained by TEM, HRTEM and selected-area electron diffraction (SAED), as shown in Fig. 4a displayed the overview of a typical NiCo_2_O_4_ nanocactus taken from the Ni substrate (Fig. 3a–c), which consisted of nanoneedles (Fig. 4b). In contrast to NiCo_2_O_4_ NCAs, NiCo_2_O_4_@NiCo_2_O_4_ NCAs had a peculiar core/shell structure with a porous NiCo_2_O_4_ shell (∼50 nm thick) surrounding a continuous core (∼40 nm in diameter) (Fig. 4c). A close examination of the shell reveals a number of hairy needles rooted in the core. The HRTEM image (Fig. 4d) presented that the lattice fringes of the shell were ∼0.247 and 0.205 nm, corresponding to the (311) and (400) planes of spinel structured NiCo_2_O_4_, respectively. The SAED pattern (Fig. 4e) showed well-defined diffraction rings, indicating the poly-crystalline nature of the cubic phase. These rings can be readily indexed to the (111), (220), (311), (400) and (422) planes of the cubic NiCo_2_O_4_ phase, which were consistent with the above XRD results. The EDS spectrum in Fig. 4f showed that the nanostructure consisted of O, Co and Ni, and the atomic ratio of Co to Ni was approximately 2:1.

**Figure 5:**
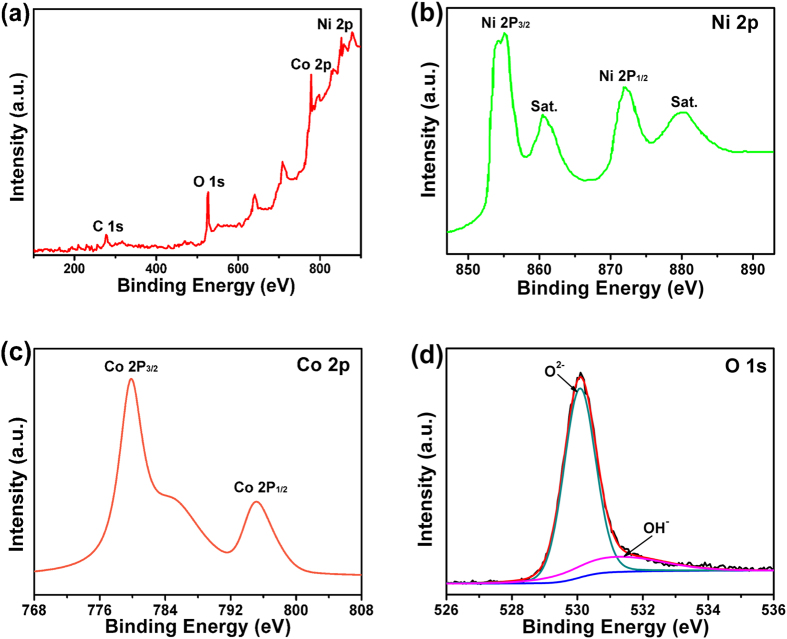
(**a**) XPS general spectrum of as-synthesized NiCo_2_O_4_@NiCo_2_O_4_ NCAs; and (**b-d**) XPS survey scan of Ni 2p, Co 2p and O 1 s regions, respectively.

The chemical compositions of the NiCo_2_O_4_@NiCo_2_O_4_ NCAs were further analyzed by X-ray photoelectron spectroscopy (XPS), as shown in Fig. 5. The general survey spectrum (Fig. 5a) indicated the presence of C, Ni, Co and O elements and the absence of other impurities. The Ni 2p spectrum (Fig. 5b) contained two prominent 2p_3/2_ and 2p_1/2_ spin-orbit peaks at binding energies of 853.5 and 872.6 eV, and two shakeup satellites (identified as “Sat.”). Two major peaks at binding energies of 779.7 and 795.2 eV were observed from the complex Co 2p curve (Fig. 5c), corresponding to the Co 2p_3/2_ and Co 2p_1/2_ spin-orbit peaks, respectively. The high-resolution O 1s spectrum (Fig. 5d) showed three peaks. Specifically, the peak at 530.0 eV is typical of metal-oxygen bonds^26^. The peak at 531.1 eV is usually associated with defects, contaminants and surface species, like hydroxyls, chemisorbed oxygen and under-coordinated lattice oxygen^27^. The peaks at ∼531.7 eV can be attributed to multiplicity of physi- and chemi-sorbed water on or near the surface^25^.

To evaluate the porous characteristics of the hierarchical NiCo_2_O_4_@NiCo_2_O_4_ NCAs, N_2_ adsorption-desorption measurements were performed. According to the IUPAC (International Union of Pure and Applied Chemistry) classifications of hysteresis loops^28^, the plots in Figure S2 exhibited type IV isotherms with type H3 hysteresis loops, indicating the existence of a typical mesoporous microstructure^29^,^30^. After a steady increase, the adsorbed nitrogen volume surged at a relative pressure close to unity, which implied the existence of large interconnection voids or void space within the nanocactuses. The pore size distribution (PSD) data (inset of Figure S2a) showed that the majority of the pores fell within the range of 3–8 nm, which was known to be optimal for energy applications^31^,^32^. The mesoporous structure resulted in a high porosity of 0.15 cm^3^ g^−1^ and a large BET specific surface area of ~115 m^2^ g^−1^. As a comparison, Figure S2b revealed a porosity of 0.11 cm^3^ g^−1^ and a small BET surface area of ~89 m^2^ g^−1^ for the NiCo_2_O_4_ NCAs. The large increase in surface area after the deposition of NiCo_2_O_4_ shell arose from the meso/microporosity of the coating.

### Electrochemical performance of SCs

**Figure 6:**
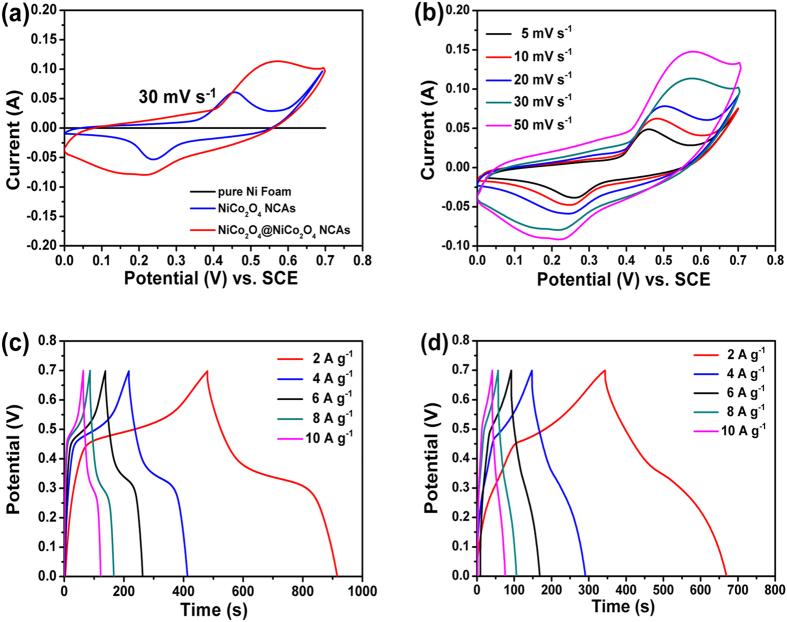
(**a**) CV curves for NiCo_2_O_4_@NiCo_2_O_4_ NCAs, NiCo_2_O_4_ NCAs and pure Ni foam, recorded at a scan of 30 mV s^−1^; (**b, c**) CV and galvanostatic charge-discharge curves of the NiCo_2_O_4_@NiCo_2_O_4_ NCAs at different scan rates and different current densities in 2 M KOH aqueous solution, respectively; (**d**) galvanostatic charge-discharge curves of the NiCo_2_O_4_ NCAs at different current densities.

Cyclic voltammetry (CV) measurements were performed to examine the electrochemical characteristics and quantify the specific capacitances of the electrodes. Figure 6a showed the CV of the NiCo_2_O_4_@NiCo_2_O_4_ NCA electrode in comparison with those of the NiCo_2_O_4_ NCA and neat Ni foam counterparts at a scan rate of 30 mV s^−1^. The signal from Ni foam was negligible compared to other CVs. The area integrated within the current-potential curve of the core/shell structured NiCo_2_O_4_@NiCo_2_O_4_ electrode was remarkably larger than that of the NiCo_2_O_4_ electrode, indicating much higher electrochemical reaction activities of the former. The synergy arising from the presence of highly porous NiCo_2_O_4_ shell with numerous hairy needles and large surface area appeared to be responsible for the enhanced ion diffusion and fast electron transfer in the NiCo_2_O_4_@NiCo_2_O_4_ electrode. It is worth noting that the redox peak positions of the two electrode materials are significantly different, possibly ascribed to the difference in electrode polarization behavior during the CV tests. The polarization behavior is closely related to the chemical composition and morphology of the electrode material. The redox reactions in an alkaline electrolyte can be expressed as follows^10^,^33^,^34^:


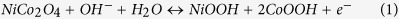



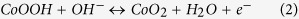


Figure 6b showed the typical CV curves of the NiCo_2_O_4_@NiCo_2_O_4_ NCAs obtained at different scan rates. The shape of the curves indicates pseudocapacitive characteristics of the NiCo_2_O_4_@NiCo_2_O_4_ electrode. There was in general one pair of broadly and poorly defined redox peaks, originating from the faradaic redox reactions related to M-O/M-O-OH, where M represents both the Ni and Co ions^35^,^36^,^37^,^38^. When the scan rate increased from 5 to 50 mV s^−1^, the corresponding current was also enhanced while the shape of the CV curves remained unchanged, except for the shifts of the peak positions. The galvanostatic charge/discharge (GCD) curves obtained at different current densities at potentials between 0 and 0.7 V were shown in Fig. 6c. With increasing current density, the curves held excellent symmetry with negligible IR drops, indicating outstanding electrochemical reversibility. As a comparison, the GCD curves of the NiCo_2_O_4_ NCA electrode were shown in Fig. 6d, exhibiting shorter discharge time.

**Figure 7:**
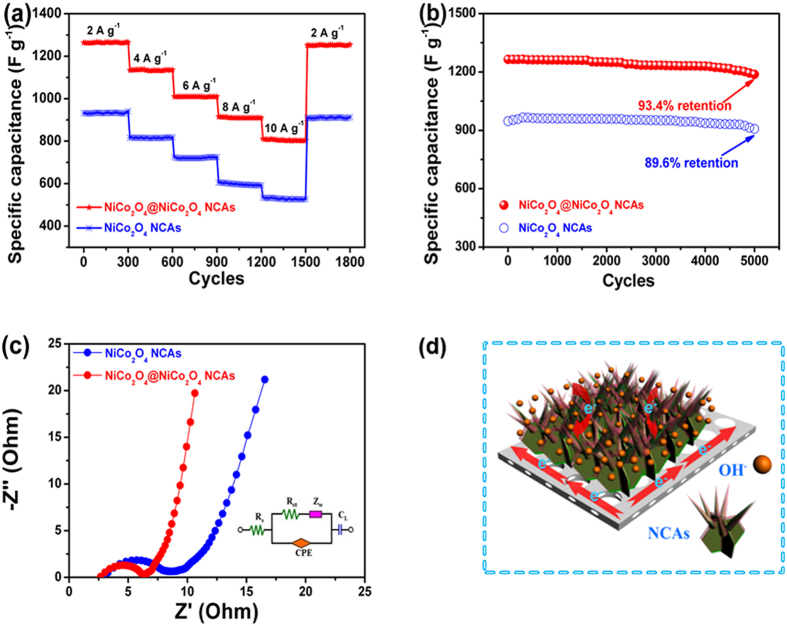
(**a**) rate capabilities of the NiCo_2_O_4_@NiCo_2_O_4_ and NiCo_2_O_4_ NCA electrodes at different current densities; (**b**) cyclic performance of the NiCo_2_O_4_@NiCo_2_O_4_ and NiCo_2_O_4_ NCA supercapacitor electrodes at a current density of 2 A g^−1^; (**c**) Nyquist plots of the NiCo_2_O_4_ and NiCo_2_O_4_@NiCo_2_O_4_ NCA electrodes and equivalent circuit (inset); (**d**) schematic representation of rechargeable supercapacitive electrode made from NiCo_2_O_4_@NiCo_2_O_4_ NCAs on Ni foam.

In line with above observations, the NiCo_2_O_4_@NiCo_2_O_4_ NCA electrode exhibited much higher capacitance and better cycle stability than the NiCo_2_O_4_ counterpart. Clearly, the NiCo_2_O_4_@NiCo_2_O_4_ electrode had higher specific capacitance over the whole current density range than the NiCo_2_O_4_ electrode, as shown in Fig. 7a. At a relatively low current density of 2 A g^−1^, the NiCo_2_O_4_@NiCo_2_O_4_ NCA electrode delivered a high capacitance of 1264 F g^−1^, which was reduced to 810 F g^−1^ with ~64% retention when the current density was increased to 10 A g^−1^. The comparison of the capacitances between the current study and similar metal oxide core/shell structured electrodes taken from the literature is shown in Table S1. The capacitance of the NiCo_2_O_4_@NiCo_2_O_4_ electrode in this study was proven to be among the best, confirming that the design of the NiCo_2_O_4_@NiCo_2_O_4_ nanocactus electrode with a hierarchical core/shell structure was efficient for supercapacitor applications. The electrode showed excellent stability without any noticeable degradation for 300 cycles at the same current density. When the current density was reverted to 2 A g^−1^, the capacitance was recovered to ~1252 F g^−1^, demonstrating excellent rate performance of the electrode. The very small loss of less than 1% of the initial value may result from the incomplete contacts between part of the unstable NiCo_2_O_4_@NiCo_2_O_4_ NCAs and the substrate, causing deteriorated electron transfer and ion diffusion. Besides rate capability, the cycle stability of SCs is another crucial parameter for practical applications. The long-term stability of the electrodes was examined at 2 A g^−1^ as shown in Fig. 7b. It was found that the NiCo_2_O_4_@NiCo_2_O_4_ electrode exhibited an excellent long-term stability of more than 93% capacitance retention after 5000 cycles, whereas the NiCo_2_O_4_ electrode showed a slightly lower 90% capacitance retention for the same cycles.

The ion diffusion and electron transfer in the two electrode materials were evaluated using the EIS measurements (Fig. 7c). The two impedance spectra were similar, all composed of one semicircle component at high frequency range and a linear component at low frequency range. The internal resistance (*R*_s_) is the sum of the ionic resistance of electrolyte, the intrinsic resistance of active materials and the contact resistance at the active material/current collector interface^39^, and can be obtained from the intercept of the plots on the real axis. The semicircle of Nyquist plot corresponds to the Faradic reactions and its diameter represents the interfacial charge transfer resistance (*R*_ct_). The inset of Fig. 7c showed an equivalent circuit used to fit the EIS curves to measure *R*_s_ and *R*_ct_, where Z_w_ and CPE are the Warburg impendence and the constant phase element, respectively^40^, and the fitting results were shown in Table S2, confirming much lower *R*_s_ and *R*_ct_ values of the NiCo_2_O_4_@NiCo_2_O_4_ electrode than the NiCo_2_O_4_ electrode. Furthermore, the NiCo_2_O_4_@NiCo_2_O_4_ electrode presented a higher slope and a shorter line in the low frequency region, suggesting faster OH^–^ diffusion rates and smaller variation of diffusion paths. The *R*_ct_ increased by 0.6 Ω after 5000 cycles confirming that the NiCo_2_O_4_@NiCo_2_O_4_ NCA nanostructures were well preserved, consistent with the very stable cyclic performance (Fig. 7b). These results demonstrated that the combination of fast ion diffusion and low electron transfer resistance resulted in enhanced electrochemical performance of the core/shell structured NCA electrode. In addition, the interconnected network of NiCo_2_O_4_@NiCo_2_O_4_ NCAs directly grown on a Ni foam substrate assured good mechanical adhesion to the underneath current collector and excellent electrical conductivities^41^. The mechanism of charge transfer in the NiCo_2_O_4_@NiCo_2_O_4_ NCA electrode is schematically shown in Fig. 7d. Along with these ameliorating structural features, the freestanding nature of the electrode allowed fast electron/ion transport without the needs of conductive additives or polymer binder which usually adds extra interfacial resistance.

### Electrochemical performance of LIBs

**Figure 8:**
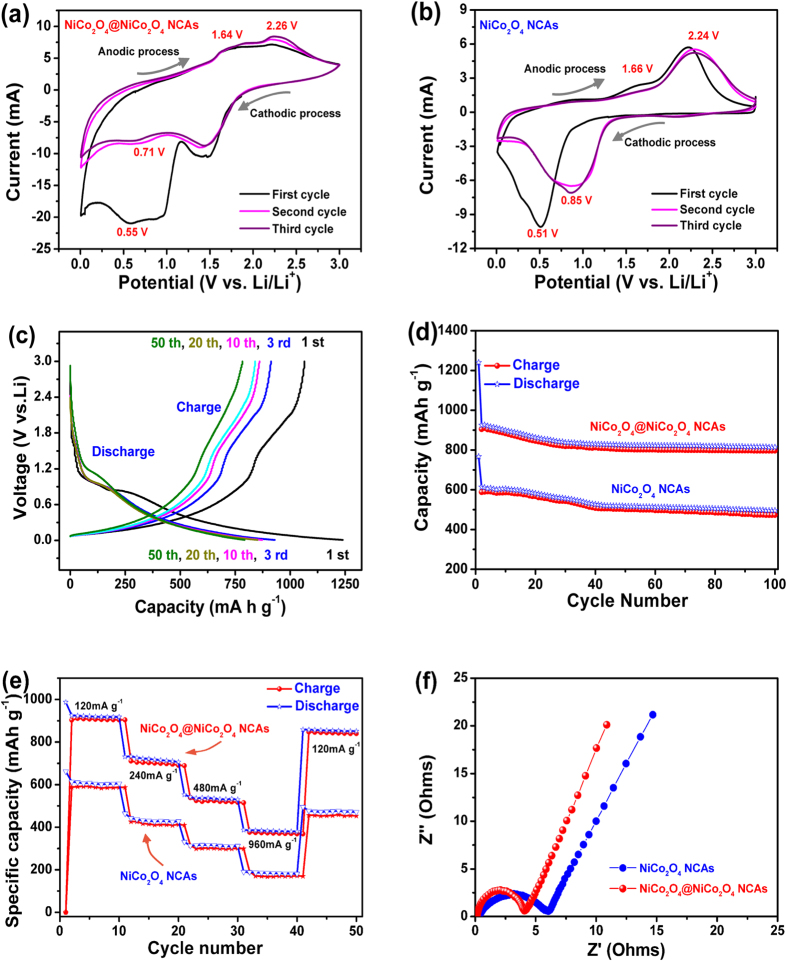
Electrochemical performance of LIBs: (a, b) CV curves of the NiCo_2_O_4_@NiCo_2_O_4_ NCA and NiCo_2_O_4_ NCA electrodes at a scan speed of 0.5 mV s^−1^ in the voltage ranging 0.01–3.0 V vs. Li; (**c**) galvanostatic discharge/charge profiles of the NiCo_2_O_4_@NiCo_2_O_4_ NCA anode at a current density of 120 mA g^−1^; (**d**) cyclic performance at 120 mA g^−1^; (**e**) rate capabilities measured at different current densities; (**f**) electrochemical impedance spectra after 100th cycles of the two anodes.

Figure 8a shows the first three CV curves of the NiCo_2_O_4_@NiCo_2_O_4_ electrode at room temperature in the range of 0.01 ~ 3.0 V versus Li/Li^ + ^at a scan rate of 0.5 mV s^−1^. Based on the previously reported storage mechanism of NiCo_2_O_4_^42^, the Li insertion and extraction reactions can be expressed as follows:


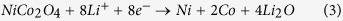



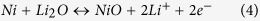



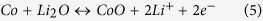



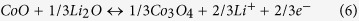


There was a strong irreversible cathodic peaks located around 0.55 V in the first cycle, corresponding to the electrochemical Li insertion reaction (eq. 3) due to the formation of Ni and Co. Well-defined anodic peaks were observed at 1.64 V and 2.26 V, indicating the extraction of Li^ + ^in the electrode materials (equations 4, 5, 6). The subsequent cycles differ slightly from the first one, indicating different redox behavior. It was worth noting that the CV peaks obtained in the 2^nd^ and 3^rd^ cycles overlapped, suggesting highly reversible electrochemical reactions taking place after the first discharge/charge cycle. The corresponding CV curves of the NiCo_2_O_4_ electrode (Fig. 8b) were dissimilar to those of the NiCo_2_O_4_@NiCo_2_O_4_ electrode, indicating different capacity performances between the two. The discharge/charge voltage profiles of the NiCo_2_O_4_@NiCo_2_O_4_ electrode (Fig. 8c) revealed a distinct plateau between 0.82 and 1.1 V. Among them, the plateau of the first discharge curve was slightly lower than the others, which is associated with the irreversible reaction of NiCo_2_O_4_ and Li^ + ^according to equation 1, in consistence with the CV results (Fig. 8b). The NiCo_2_O_4_@NiCo_2_O_4_ NCAs presented a high initial capacity of 1240 mA h g^−1^ which was reduced to 925 mA h g^−1^ in the third cycle, resulting in a first-cycle coulombic efficiency of ~75%. The abrupt capacity loss is most likely due to the irreversible reactions by the formation of the solid electrolyte interface (SEI) layer, as seen also from the shape difference between the discharge voltage profiles in Fig. 8a^43^.

Figure 8d presents excellent cyclic stability of the two electrodes when measured at a current density of 120 mA g^−1^. The NiCo_2_O_4_@NiCo_2_O_4_ electrode delivered a specific capacity 925 mA h g^−1^ after the 2^nd^ cycle, which was reduced to 830 mA h g^−1^ after the 100th cycle with a Coulombic efficiency of 90%. This value is slightly higher than 86% of the NiCo_2_O_4_ electrode. Nevertheless, the capacities of the former electrode were consistently higher than the latter electrode by more than 300 mA h g^−1^ over the whole cycles studied. The relatively lower charge transfer resistance of the former electrode than the latter (Fig. 8f) is partly responsible. The rate performance of both the electrodes was evaluated at different current densities (Fig. 8e). Both the NiCo_2_O_4_@NiCo_2_O_4_ and NiCo_2_O_4_ electrodes were first cycled at a current density of 120 mA g^−1^. Irreversible capacity losses during the initial two cycles were observed for both the electrodes presumably due to decomposition of the electrolyte and/or solvent. Nevertheless, the first discharge capacity of ~925 mA h g^-1^ for the NiCo_2_O_4_@NiCo_2_O_4_ electrode was much higher than ~605 mA h g^−1^ for the NiCo_2_O_4_ electrode. Subsequently, the current density was increased stepwise to 960 mA g^−1^, and the resulting specific capacities were ~407 and 212 mA h g^−1^, respectively. After the current density was reverted to 120 mA g^−1^ following 40 cycles, the capacity was recovered to ~876 mA h g^−1^ for the NiCo_2_O_4_@NiCo_2_O_4_ electrode, which was much higher than 480 mA h g^−1^ the NiCo_2_O_4_ counterpart under the same condition. The excellent rate performance and cyclability render the NiCo_2_O_4_@NiCo_2_O_4_ electrode a very promising candidate for LIB application.

To gain further insight into the relative performance of the electrodes, the EIS spectra of the two electrode materials were measured after 100 cycles, as shown in Fig. 8f. The Nyquist plots were typically in the form of an arc at high frequencies and a straight line with ~45^o^ in slope at low frequencies. It was obvious that both the NiCo_2_O_4_@NiCo_2_O_4_ and the NiCo_2_O_4_ NCAs had similar diffusion resistance while the former revealed a relatively lower charge transfer resistance than the latter. These observations are considered consistent with the better specific capacities, cyclic stability and rate performance of the NiCo_2_O_4_@NiCo_2_O_4_ electrode than the NiCo_2_O_4_ counterpart.

## Conclusion

In summary, we have demonstrated the rational design and fabrication of the 3D hierarchical porous NiCo_2_O_4_@NiCo_2_O_4_ core/shell structures through a facile and low-cost approach. The smart electrode made of these NiCo_2_O_4_@NiCo_2_O_4_ core/shell NCAs exhibited excellent electrochemical performance both in SCs and LIBs in terms of specific capacity/capacitance, cyclic stability and rate performance. These properties were much better than those of the NiCo_2_O_4_ NCA structure.

Several unique structural features and ameliorating properties are considered responsible for the excellent electrochemical performance of the NiCo_2_O_4_@NiCo_2_O_4_ core/shell NCA electrode. (i) The large surface area with hairy needles and hierarchical mesoporosity of the shell enabled full exposure of the active material to the electrolyte. The high porosity means that the core materials were also accessible to the electrolyte for energy storage. (ii) The open geometry between the NCAs allowed easy penetration of the electrolyte into the inner region of the electrode, increasing the utilization of the active materials. (iii) The Ni foam functioned as a highly conductive and rigid substrate, making it possible to prepare freestanding electrodes without using conductive additives or polymer binders, which also significantly contributed to their enhanced electrochemical performance. The rigid substrate may also help maintain the structural integrity of the NiCo_2_O_4_ core during charge/discharge cycles.

## Methods

### Synthesis of mesoporous NiCo_2_O_4_ nanocactus arrays (NiCo_2_O_4_ NCAs) on nickel foam

All the reagents were analytical grade and directly used after purchase without further purification. Prior to deposition, nickel foam of 1.5 cm × 4.0 cm in rectangular shape were cleaned by sonication sequentially in acetone, 1 M HCl solution, deionized water, and ethanol for 15 min each. NiCo_2_O_4_ NCAs were grown on Ni foam via a simple one-pot hydrothermal process. 1 mmol (0.24 g) of NiCl_2_**·**6H_2_O and 2 mmol (0.48 g) of CoCl_2_·6H_2_O were dissolved into 35 mL of deionized (DI) water and 5 mL of ethanol absolute, followed by the addition of 15 mmol (0.90 g) of urea and 6 mmol (0.22 g) of NH_4_F at room temperature, and the mixture was stirred to form a clear pink solution. Then the mixture was transferred into a 50 mL Teflon-lined stainless steel autoclave. The cleaned Ni foam was immersed in the mixture, and the autoclave was kept at 120 ^o^C for 3 h. After cooling down to room temperature, the Ni foam was taken out and washed with DI water and ethanol several times to obtain NiCo_2_O_4_ precursor on its surface. The as-prepared NiCo_2_O_4_ precursor grown on the Ni substrate was annealed at 350 ^o^C in air for 2 h with the temperature rising at a ramping rate of 1 ^o^C min^−1^ to obtain NiCo_2_O_4_ nanocactus arrays.

### Preparation of 3D NiCo_2_O_4_@NiCo_2_O_4_ hierarchical structures (NiCo_2_O_4_@ NiCo_2_O_4_ NCAs) on nickel foam

The NiCo_2_O_4_ nanocactus arrays on Ni foam substrate were used as the scaffold for further growth of the NiCo_2_O_4_ shell structure by electrochemical deposition. The electrodeposition was performed in a standard three-electrode glass cell at 25 °C, using NiCo_2_O_4_ NCAs as the working electrode, saturated calomel electrode (SCE) as the reference electrode, and a Pt foil as the counter electrode. The electrolyte Co_2*x*_Ni_x_(OH)_2_ was prepared from 70 ml of 0.1 M metal ion solution at a Ni^2 + ^/Co^2 + ^concentration ratio of 1:2. Co_x_Ni_1-x_(OH)_2_ acicular needles were deposited on the NiCo_2_O_4_ NCAs by the potential static of −1.0 V for 10 min. The substrate was taken off and rinsed with DI water and ethanol under ultrasonication, and dried in air. Then, the substrate was calcined at 350 °C for 2 h in a furnace to convert Co_x_Ni_1-x_(OH)_2_ to NiCo_2_O_4_ shell on the NiCo_2_O_4_ NCA core.

### Materials characterizations

The crystalline structure and phase purity of the products were identified by X-ray diffraction (XRD) using a D8 Advance (Germany, Bruker) automated X-ray diffractometer system with Cu-Kα (λ = 1.5418 Å) radiation at 40 kV and 40 mA ranging from 10° to 80° at room temperature. Raman spectroscopy was carried out using an INVIA Raman microprobe (Renishaw Instruments, England) with a 532 nm laser source and a 50 × objective lens. The morphologies and microstructures were characterized by field emission scanning electron microscopy (FSEM, JEOL S-4800) and transmission electron microscopy (TEM, JEOL JEM-2010). The elemental analysis was carried out using a Bruker-QUANTAX, energy-dispersive X-ray spectroscope (EDS) attached to a FESEM. X-ray photoelectron spectroscopy (XPS) was conducted on a modified PHI 5600 XPS system. The Brunauer-Emmett-Teller (BET) surface areas of the electrode materials of 20 mg in weight were determined by the nitrogen sorption/desorption measurement at 77 K conducted on a Quadrasorb SI-MP Surface Area and Porosity Analyzer (American, Quantachrome).

### SC performance measurements

The electrochemical measurements were performed on an electrochemical workstation (CHI 660D, CH Instruments Inc., Shanghai) using a three-electrode mode in 2 M KOH aqueous solution within the potential window of approximately 0 to 0.7 V. The NiCo_2_O_4_@NiCo_2_O_4_ hybrid or pristine NiCo_2_O_4_ NCAs (S_NiCo2O4@NiCo2O4_ ≈ 1 × 3 cm^2^; m_hydrothermal_ ≈ 15.0 mg, m_electrodeposition_ ≈ 5.0 mg) were used directly as the working electrode. A Pt foil and a standard calomel electrode (SCE) were used as the counter electrode and the reference electrode, respectively. The electrochemical impedance spectroscopy (EIS) was performed by applying an AC voltage with 10 mV amplitude in the frequency range from 0.01 Hz to 100 kHz. The galvanostatic charge-discharge tests were conducted on a LAND battery program-control test system. The specific capacitance, C (F g^−1^), was calculated according to the following equation:


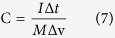


Where *I* (mA) represents the discharge current, and *M* (mg), Δv (v) and Δ*t* (s) designate the mass of active material, potential drop during discharge and total discharge time, respectively.

### Battery performance measurements

Electrochemical tests of LIBs were carried out using CR2032 coin cells. The cells were assembled in an Argon-filled glovebox (Mbraun, Unilab, Germany) using NiCo_2_O_4_@NiCo_2_O_4_ NCAs (m_NiCo2O4@NiCo2O4_ ≈ 8 mg; m_hydrothermal_: m_electrodeposition_ = 3 : 1) and NiCo_2_O_4_ NCAs (≈6 mg) as the working electrode, a Li-metal circular foil (0.61 mm thick) as the counter and reference electrode, a microporous polypropylene membrane as the separator. The solution consisting of 1 M LiPF_6_ in ethylene carbonate (EC)/diethyl carbonate (DEC) (1: 1 by volume) was used as the electrolyte. The cells were aged for 10 h before the measurements to ensure full penetration of the electrolyte to the electrodes. The discharge and charge measurements were carried out on an Arbin battery test system (BT2000) in the voltage window of 0.01 ~ 3.0 V at different current densities. The cyclic voltammogram (CV) tests were performed on a multichannel battery tester (model SCN, USA) at a scan rate of 0.5 mV s^−1^.

## Additional Information

**How to cite this article**: Cheng, J. *et al.* Hierarchical Core/Shell NiCo_2_O_4_@NiCo_2_O_4_ Nanocactus Arrays with Dual-functionalities for High Performance Supercapacitors and Li-ion Batteries. *Sci. Rep.* **5**, 12099; doi: 10.1038/srep12099 (2015).

## Supplementary Material

Supplementary Information
