# Supplementary material for: Hierarchical Core/Shell NiCo2O4@NiCo2O4 Nanocactus Arrays with Dual-functionalities for High Performance Supercapacitors and Li-ion Batteries
Source: Sci Rep. 2015 Jul 1;5:12099. doi: 10.1038/srep12099 (PMC4487229; doi:10.1038/srep12099)
Supplement: Supplementary Information [file srep12099-s1.docx]

**Supporting Information**

**Hierarchical Core/Shell NiCo_2_O_4_@NiCo_2_O_4_ Nanocactus Arrays with Dual-functionalities for High Performance Supercapacitors and Li-ion Batteries**

Jinbing Cheng^1,6^, Yang Lu^1,2,6^, Kangwen Qiu^1,6^, Hailong Yan^1^, Jinyou Xu^1^, Lei Han^1^, Xianming Liu^3^, Jingshan Luo^4^, Jang-Kyo Kim^5^ & Yongsong Luo^1^

^1^Key Laboratory of Advanced Micro/Nano Functional Materials, School of Physics and Electronic Engineering, Xinyang Normal University, Xinyang, P. R. China;

^2^School of Material Science and Engineering, Hebei University of Technology, Tianjin, P. R. China;

^3^College of Chemistry and Chemical Engineering, Luoyang Normal University, Luoyang, P. R. China;

^4^Division of Physics and Applied Physics, School of Physical and Mathematical Sciences, Nanyang Technological University, Singapore;

^5^Department of Mechanical and Aerospace Engineering, The Hong Kong University of Science and Technology, Clear Water Bay, Kowloon, Hong Kong, P. R. China

^6^These authors contributed equally to this work.

Correspondence: Professor Yongsong Luo, Key Laboratory of Advanced Micro/Nano Functional Materials, School of Physics and Electronic Engineering, Xinyang Normal University, Xinyang, P. R. China

E-mail address: ysluo@xynu.edu.cn


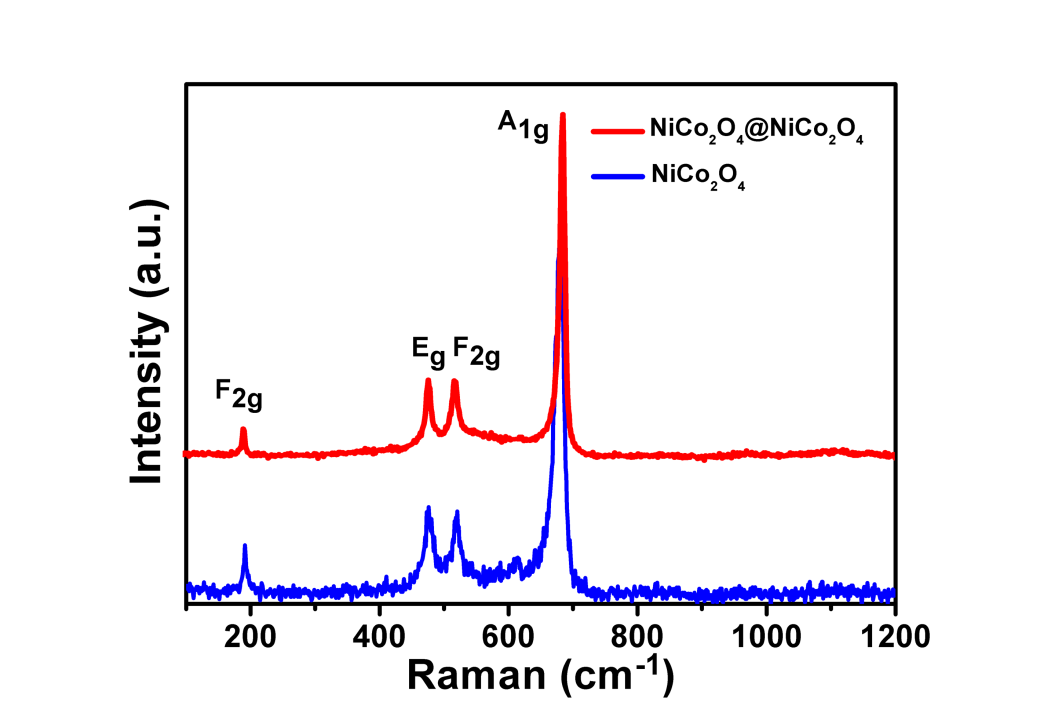


**Figure S1** Raman spectra of NiCo_2_O_4_@NiCo_2_O_4_ and NiCo_2_O_4_ NCAs.


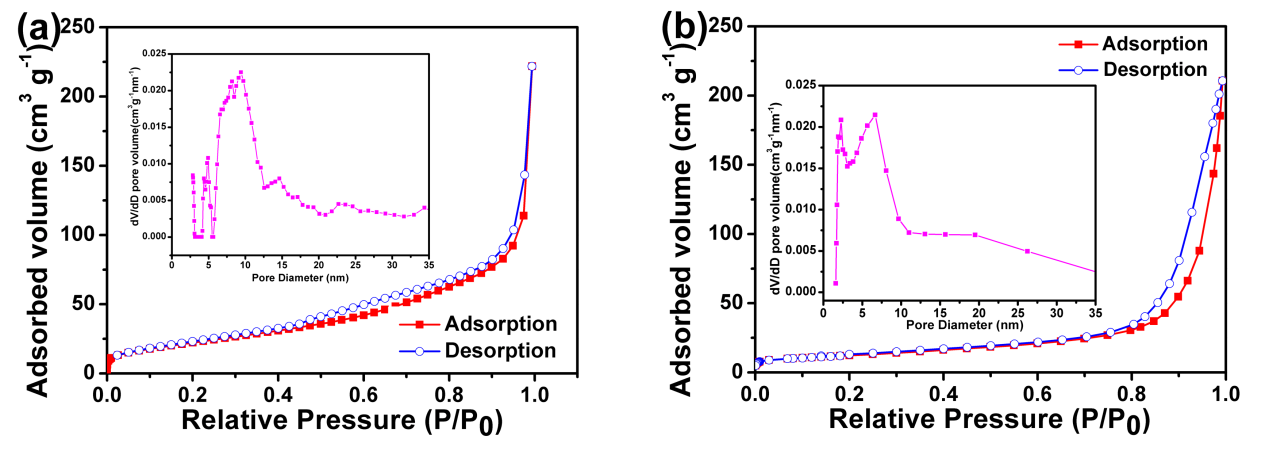


**Figure S2** Nitrogen adsorption/desorption isotherms and pore-size distribution curves for (a) NiCo_2_O_4_@NiCo_2_O_4_ and (b) NiCo_2_O_4_ NCAs.

**Table S1** Comparison of specific capacitances of NiCo_2_O_4_@NiCo_2_O_4_ core/shell structured electrodes with different nanostructured electrodes reported in literature.

| **Electrode material** | **Current density** | **Specific capacitance** | **Reference** |
| --- | --- | --- | --- |
| NiCo_2_O_4_@NiCo_2_O_4_ nanocactus | 2 A g^-1^ | 1264 F g^-1^ | Current study |
| NiCo_2_O_4_ nanocactus | 2 A g^-1^ | 932 F g^-1^ | Current study |
| MnCo_2_O_4_ nanowire | 2 A g^-1^ | 350 F g^-1^ | 1 |
| MnMoO_4_@CoMoO_4_ nanowire | 2 A g^-1^ | 163 F g^-1^ | 2 |
| Co_3_O_4_@NiO nanowire | 2 A g^-1^ | 853 F g^-1^ | 3 |
| ZnO@MnO_2_ nanorod | 2 A g^-1^ | 501 F g^-1^ | 4 |
| CuCo_2_O_4_ nanoparticle | 2 A g^-1^ | 280 F g^-1^ | 5 |
| NiCo_2_O_4_ nanosphere | 2 A g^-1^ | 660 F g^-1^ | 6 |
| NiCo_2_O_4_@MnO_2_ nanoflake | 2 A g^-1^ | 923 F g^-1^ | 7 |

**References**

1. Li, L. et al. One-dimension MnCo_2_O_4_ nanowire arrays for electrochemical energy storage. *Electrochim. Acta* **116**, 467-474 (2014).

2. Mai, L.-Q. et al. Hierarchical MnMoO_4_/CoMoO_4_ heterostructured nanowires with enhanced supercapacitor performance. *Nat. Commun*. **2**, 381 (2011).

3. Xia, X. et al. High-quality metal oxide core/shell nanowire arrays on conductive substrates for electrochemical energy storage. *ACS Nano* **6**, 5531-5538 (2012).

4. Li, S. et al. Three-dimensional MnO_2_ nanowire/ZnO nanorod arrays hybrid nanostructure for high-performance and flexible supercapacitor electrode. *J. Power Sources* **256**, 206-211 (2014).

5. Pendashteh, A., Rahmanifar, M. S., Kanerc, R. B. & Mousavi, M. F. Facile synthesis of nanostructured CuCo_2_O_4_ as a novel electrode material for high-rate supercapacitors. *Chem. Commun*. **50**, 1972-1975 (2014).

6. Yuan, C. et al. Template-engaged synthesis of uniform mesoporous hollow NiCo_2_O_4_ sub-microspheres towards high-performance electrochemical capacitors. *RSC Adv*. **3**, 18573-18578 (2013).

7. Li, G. Sponge-like NiCo_2_O_4_/MnO_2_ ultrathin nanoflakes for supercapacitor with high-rate performance and ultra-long cycle life. *J. Mater. Chem. A* **2**, 7738-7741 (2014).

**Table S2** Internal resistance (*R*_s_) and charge transfer resistance (*R*_ct_) of synthesized composites.

| **Materials** | ***R*_s_ (**Ω**)** | ***R*_ct_ (**Ω**)** | ***R*_ct_ (**Ω**) after 5000 cycles** |
| --- | --- | --- | --- |
| NiCo_2_O_4_ NCA | 2.40 | 5.12 | 6.07 |
| NiCo_2_O_4_@NiCo_2_O_4_ NCA | 2.23 | 3.81 | 4.41 |
